# Supplementary material for: Identification of photoperiod-induced specific miRNAs in the adrenal glands of Sunite sheep (Ovis aries)
Source: Front Vet Sci. 2022 Jul 22;9:888207. doi: 10.3389/fvets.2022.888207 (PMC9354845; doi:10.3389/fvets.2022.888207)
Supplement: Supplementary file 1 [file Data_Sheet_1.ZIP › Supplementary file/miRDeep2 file.zip!/miRDeep2 file/Novel_Structure/SP42A2/Novel_572.pdf]

Provisional ID	:	NC_019479.2_37237
Score total	:	1.8
Score for star read(s)	:	-1.3
Score for read counts	:	0
Score for mfe	:	1.5
Score for randfold	:	1.6
Score for cons. seed	:	
Total read count	:	88
Mature read count	:	88
Loop read count	:	0
Star read count	:	0

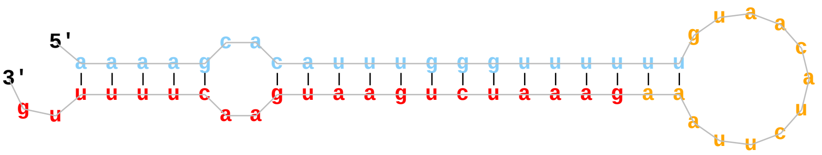

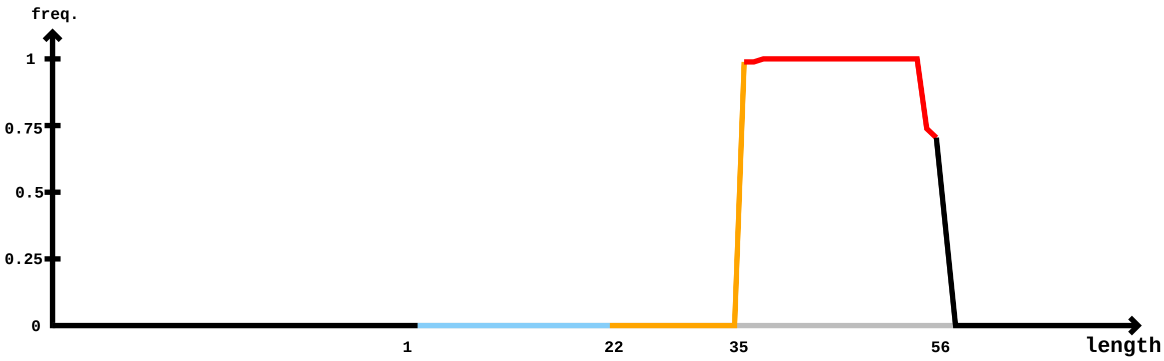


```
                                                                     Star                                 Mature
5'- accaaaauuacagaggcuaauuuauugguuggcuaaaaagcacauuuggguuuuuuguaacaucuuaaagaaaucugaaugaacuuuuugggcaacccaauacauucuaa   -3'    exp
    ......................((((((((((.((((((((..((((((((((((((..........))))))))))))))..)))))))).))))))))))........     reads    mm    sample
    .....................................................................Aaaaucugaaugaacuuuu......................     23       1     seq
    .....................................................................gaaaCcugaaugaacuuuuu.....................     2        1     seq
    .....................................................................Aaaaucugaaugaacuuuuug....................     57       1     seq
    .....................................................................gaaaucugaaugaacuuuuug....................     1        0     seq
    .....................................................................gaaaCcugaaugaacuuuuugg...................     3        1     seq
    .....................................................................gaaaucugaaugaacuuuuugg...................     1        0     seq
    .......................................................................aaucugaaugaacuuuuu.....................     1        0     seq
```
